# Dysarthria detection based on a deep learning model with a clinically-interpretable layer

**DOI:** 10.1121/10.0016833

**Published:** 2023-01-10

**Authors:** Lingfeng Xu, Julie Liss, Visar Berisha

**Affiliations:** 1 School of Computing and Augmented Intelligence, Arizona State University, Tempe, Arizona 85281, USA; 2 College of Health Solutions, Arizona State University, Tempe, Arizona 85281, USA lingfen3@asu.edu; julie.liss@asu.edu; visar@asu.edu

## Abstract

Studies have shown deep neural networks (DNN) as a potential tool for classifying dysarthric speakers and controls. However, representations used to train DNNs are largely not clinically interpretable, which limits clinical value. Here, a model with a bottleneck layer is trained to jointly learn a classification label and four clinically-interpretable features. Evaluation of two dysarthria subtypes shows that the proposed method can flexibly trade-off between improved classification accuracy and discovery of clinically-interpretable deficit patterns. The analysis using Shapley additive explanation shows the model learns a representation consistent with the disturbances that define the two dysarthria subtypes considered in this work.

## 1. Introduction

Dysarthria is a motor speech disorder caused by impaired neuromuscular control (Enderby, 2013) and can lead to severe impact on speech quality and intelligibility. Effective detection of dysarthria has the potential to help neurologists in detecting early signs of several neurological diseases like Parkinson's disease (PD), amyotrophic lateral sclerosis (ALS), and Huntington's disease (HD), tracking their progression and optimizing their treatment (Stone *et al.*, 2005).

The current clinical standard for the detection of dysarthria is perceptual evaluation (i.e., listening) by speech language pathologists. Despite the availability of standardized assessment protocols to ensure the reliability of perceptual evaluation (Enderby, 1980), there still exists inter-rater and intra-rater reliability issues, thereby complicating the interpretation of evaluation results (Carmichael and Green, 2004). More recently, several studies have proposed objective assessment of dysarthria (and of the neurological conditions that lead to dysarthria) *via* speech analytics and machine learning. For instance, Vogel *et al.* (2012) analyzed the speech signal produced by early-stage HD patients and identified a significant correlation between the abnormal speech rate and the burden of disease scores. Hazan *et al.* (2012) trained a Support Vector Machine (SVM) to detect early to mild PD speakers from different countries. Stegmann *et al.* (2020) computed the speech rate and articulatory precision features from ALS samples and validated them as sensitive metrics in tracking the longitudinal speech changes caused by ALS. Recently, deep neural networks (DNNs) have played an increasingly important role in dysarthria detection, as they do not require intensive feature engineering and show higher efficiency in capturing the various characteristics of dysarthric speech from raw inputs (Gupta *et al.*, 2021). Briefly, DNNs transform the input into latent representation embeddings that highlight the subtle hidden patterns of deficit and steer the model to detect dysarthria accurately. Models based on DNNs report higher accuracy in classifying dysarthric speakers and healthy controls in the early stages of disease (An *et al.*, 2018) and predicting the severity of dysarthria (Bhat *et al.*, 2017).

Despite their success in prediction, DNNs' complex structures make it challenging to interpret the learned representations or determine why they arrive at a particular decision, which is important in clinical applications (Kale *et al.*, 2015). An interpretable model can not only enhance its acceptance among primary care physicians but also offer more actionable insights to clinical experts, resulting in improved patient care (Che *et al.*, 2015). There has been increased interest in studying deep learning model interpretability (Zhang and Zhu, 2018). However, most of the current studies either construct “wrapper” explainer models to summarize the statistics of the original model's predictions or focus on explaining which input features are important for the DNN's final decision. For the former, adding another explainer may introduce further distrust as the explainer can never reproduce the actual decision logic of the original model (Rudin, 2019). For the latter, the input must be understandable to humans to obtain interpretability (Hohman *et al.*, 2019). For example, in computer vision, the input to a model is an image; images are human-readable therefore regions of interest in the input can be used to explain predictions made by the model. When it comes to dysarthria evaluation, the input features are speech spectrograms, which are not readily understood by clinicians as they do not map neatly to perceptual features of speech. In this circumstance, the region of interest may no longer lead to meaningful interpretation.

While most of the existing work focusing on artificial intelligence (AI) model interpretability in clinical applications assumes that the input is easily understood by users, there are two notable exceptions to this. Sturm *et al.* (2016) trained a fully connected network to do electroencephalogram (EEG) based motor imagery classification. Multi-channel EEG sequences were taken as input and layer-wise relevance propagation (LRP) was used to compute the relevance of each input data point to the classification decision. Then, the relevance vectors were averaged for each channel and projected to the scalp map, creating a heatmap that reflects the relative importance of each brain region to the motor imagery task. The paper provides an EEG-specific solution that bridges the gap between an input that is not readily understood by most clinicians (the raw EEG signal) and one that is (heatmap overlaid on the brain). In a study more directly related to speech, Tu *et al.* (2017) trained a model to predict the severity of dysarthric speech from the input signal. To enforce interpretability, the authors impose an interpretable bottleneck layer and use transfer learning to jointly learn the clinically-interpretable labels (in their case, the perceptual labels by speech-language pathologists) in the bottleneck layer and the final diagnostic label. The proposed model was able to generate an intermediate output that not only led to a more accurate dysarthria assessment but also justified the prediction by showing high correlations with the interpretable bottleneck features.

In this work, we extend the interpretable bottleneck approach proposed by Tu *et al.* (2017), with several new contributions. We train a DNN to classify 74 dysarthric patients from 91 healthy controls and constrain the model so that it also represents four interpretable acoustic features that characterize the constellation of symptoms for a given speaker. We focus on four principal acoustic features that appear across dysarthria: articulatory precision, consonant-vowel (CV) transition precision, hypernasality, and vocal quality. The focus on acoustic features is an important extension of the original work (Tu *et al.*, 2017), where they rely on *perceptual* labels provided by speech-language pathologists in the interpretable layer. A second important contribution is the introduction of Shapley additive explanation (SHAP), a unified model interpretation tool capable of computing feature importance from each instance (Lundberg and Lee, 2017). We apply SHAP to further analyze the contribution of each acoustic feature in the interpretable layer to the final prediction on both a global level (i.e., across all speakers) and at the individual speaker level. The results indicate that the proposed model can be flexibly tuned to prioritize dysarthria classification performance or clinical interpretability. When steering the model to focus on classification, it shows improved performance relative to three baselines: an eXtreme Gradient Boosting (XGBoost) model trained using the four interpretable acoustic features, a DNN trained without an interpretable bottleneck layer, and a DNN that only relies on the input mel-spectrogram to make a classification. When steering the model to focus on interpretability, the model can reveal a clinically meaningful pattern of deficit that drives the DNN to detect dysarthria and can provide insight into clinicians as to why the DNN made a particular classification decision.

## 2. Methods

### 2.1 Dataset and feature extraction

The dataset used in this study was collected at Arizona State University as part of a larger investigation on dysarthric speech. It consists of 74 dysarthric patients (age 68.14 ± 10.70 years, 31 females) with symptoms covering four dysarthria subtypes: hypokinetic (*n* = 54), ataxic (*n* = 15), mixed spastic-flaccid (*n* = 2), non-specific (*n* = 3), and 91 healthy controls (age 56.99 ± 23.80 years, 53 females). During the data acquisition process, each dysarthric patient was asked to read 20 short phrases and five sentences in four conditions: habitual (everyday speech pattern), clear, loud, and slow. The four conditions were elicited to generate multiple samples per speaker, each with different acoustic profiles. These conditions also reflect common intervention strategies that are intended to improve speech intelligibility in dysarthria. Eliciting a range of speech intelligibility from the same speaker promotes sensitivity to subtle changes in speech, which could benefit early detection. The same experiment was also conducted on healthy controls, but they were asked to read 40 phrases and five sentences. As a result, 100 speech samples (25 samples × 4 conditions) were collected from each dysarthric patient and 180 samples (45 samples × 4 conditions) from each healthy control, adding up to 23 780 individual speech samples in total.

In the feature extraction stage, the speech samples were down-sampled to 16 kHz and four acoustic features were extracted, namely, Goodness of Pronunciation (GOP) (Witt and Young, 2000), Objective Articulation Measure (OAM) (Mathad *et al.*, 2022), Objective Hypernasality Measure (OHM) (Mathad *et al.*, 2021), and Cepstral Peak Prominence (CPP) (Hillenbrand *et al.*, 1994). This collection of features characterizes four aspects of dysarthria: articulatory precision, CV transition precision, hypernasality, and vocal quality respectively. The phoneme-level GOPs were generated using a pre-trained DNN-Hidden Markov model (DNN-HMM, Tu *et al.*, 2018) and averaged to produce sample-level GOP score. The sample-level OAM and OHM were computed using the pre-trained DNN models offered by the original developers. The sample-level CPP was generated by *Praat* with default settings. This feature has been used as an overall measure of vocal quality, with studies showing that it is particularly suitable for measuring breathiness in voice (Fraile and Godino-Llorente, 2014).

Since the computation process of several of the acoustic features (GOP, OAM, OHM) relies on phoneme-level information, and a single sample may not contain all phonemes, we further averaged the features across all samples spoken by a given speaker so the influence of all phonemes was taken into account. The resulting four speaker-level values were used as interpretable labels and assigned to all samples that belonged to that speaker. Then, the features were *z*-normalized across the entire dataset to zero mean and unit standard deviation. In addition to the interpretable features, a mel-spectrogram was extracted from the first 1.25 s of each sample, the length of the shortest utterance, and this served as input to the proposed DNN model. Constraining the input to 1.25 s allowed us to make uniform the input to the DNN across all samples, and helped decrease the risk of overfitting brought about by high dimensional input (Berisha *et al.*, 2021). The signal was analyzed using a Hanning window of 20 ms with a hop length of 5 ms, and the number of mel filterbanks was 40. The extracted mel-spectrogram was of dimension 40 × 251, and the mel filterbank features were *z*-normalized for each condition (habitual, clear, loud, slow).

### 2.2 Proposed model

**Fig. 1.**
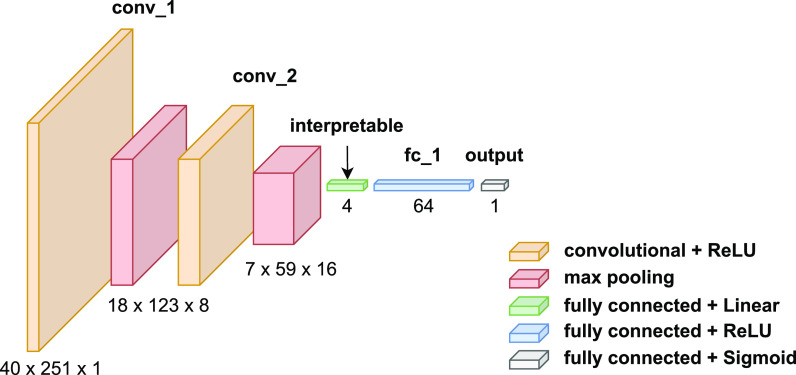
The structure of the proposed DNN model. The 4-unit layer is placed between the convolutional layers and a larger fully connected layer to both learn the features characterizing different aspects of dysarthria and to function as a bottleneck feature extractor.

The structure of the proposed DNN model is shown in Fig. 1. To begin with, the input mel-spectrogram is processed by two convolutional layers, where eight filters of shape 5 × 5 and 16 filters of shape 5 × 5 are used, respectively. Then, the output is flattened and fed into a 4-unit interpretable layer. The units in the interpretable layer are trained to learn the acoustic features that characterize different aspects of dysarthria interpretable to clinicians. The idea behind the architecture is that clinicians can interrogate the interpretable layer to better understand the model's final decision. In addition to providing a means of better understanding the decision of the model, this layer also serves as a bottleneck feature extractor, which is placed before a larger layer to constrain the solution space and improve the accuracy of the model (Yu and Seltzer, 2011). Following the interpretable layer, the output is linearly activated and fed into the next two fully connected layers. The final output is activated by a sigmoid function to classify between dysarthric and healthy speech.

### 2.3 Model training and evaluation

The proposed model was built in PyTorch and trained using a multitask training strategy; that is, the model jointly learns the four clinically-interpretable features in the bottleneck layer and the final classification label. The mean squared error (MSE) and binary cross-entropy (BCE) were taken as the loss functions of the regression task (interpretable layer task) and classification task, respectively. The final loss function was obtained by performing a weighted sum of the losses for each individual task,

$$L_{\mathrm{total}}=w\cdot L_{\mathrm{BCE}}+(1-w)\cdot L_{\mathrm{MSE}},$$ (1)

where *w* is a weight parameter that balances the model's focus on the two tasks. When *w* = 1, the model only focuses on the classification task, with no attention paid to modeling the clinically-interpretable layer. When *w* = 0, the proposed model disregards the classification task and solely focuses on learning the interpretable features. It is expected that with appropriate training and *w* value selection, we can obtain a model that not only succeeds in classifying between dysarthric and healthy speech, but also provides an intermediate layer in which each unit represents an aspect of dysarthria interpretable to clinicians.

Before training the proposed model, we established several baselines for comparison. First, we train an XGBoost model (Chen and Guestrin, 2016) using the speaker-level interpretable features as input. XGBoost is a robust implementation of Gradient Boosted Decision Trees (GBDT) (Friedman, 2001), which construct a strong classifier by combining a sequence of weak decision tree learners, each trained to minimize the errors of the previous ones. As one of the most used implementations of GBDT, XGBoost is fast during tree construction, has an improved split finding strategy, and better generalizability (Dev and Eden, 2019). We refer to it as *Baseline 1*. We use tenfold cross-validation to evaluate its performance. In each fold, 90% of the speakers were taken for training, while the remaining 10% were used for testing. During the optimization stage, a grid search was adopted to fine-tune several hyper-parameters (learning rate, the number of trees, the maximum tree depth, the minimum child weight, and Gamma), and early stopping was adopted to prevent overfitting. The training would terminate once the test metric (area under the curve, in our case) failed to improve for 10 epochs. The second baseline (*Baseline 2*) is similar in structure to the proposed model but is without the bottleneck interpretable layer. The third baseline (*Baseline 3*) is identical in structure to the proposed model, but *w* is fixed to 1, in which case the model solely focuses on the classification task and the clinically-interpretable layer is reduced to a bottleneck feature extractor. Both *Baseline 2* and *Baseline 3* were trained based on the extracted mel-spectrogram with a speaker-level tenfold cross-validation. The optimization was performed using the Adam optimizer, while the learning rate was initialized to 0.001 and decreased by 0.8 times every 10 epochs. Again, early stopping was adopted to prevent overfitting. The training would terminate once the testing loss failed to improve for 10 epochs. The classification accuracy was computed both at the sample level and speaker level. For speaker-level decisions, a majority vote strategy was adopted; that is, a speaker was classified as dysarthric when more than half of their samples were classified as dysarthric.

When training the proposed model, we used the same experimental settings as the DNN baselines. After each fold of cross-validation, the outputs of the interpretable layer were averaged at the speaker level, and two correlation coefficients, namely, the Pearson correlation coefficient (PCC) and Spearman correlation coefficient (SCC), were calculated between the speaker-level interpretable layer output and the corresponding speaker-level interpretable features. The resulting PCCs and SCCs were further averaged across the ten folds of cross-validation to obtain a general assessment on how well the model predicts the labels of the interpretable layer. The weight parameter *w* was modified to show how it impacts the balance between classification accuracy and a clinically-meaningful bottleneck representation.

### 2.4 Understanding the model's decision with SHAP values

Shapley values have been proposed as a tool for better understanding predictive models in healthcare applications (Pandl *et al.*, 2021). However, to the best of our knowledge, they have not been used in speech-based clinical models owing to a lack of input features that are interpretable. Shapley values have roots in game theory, where they were originally proposed as a means of fairly attributing the contribution of players to the result of a cooperative game (Winter, 2002). Recently, Lundberg and Lee (2017) combined Shapley value with several model interpretation tools and proposed SHAP, a framework for computing the relative importance of each input variable to the output of a machine learning model. Since the method provides information about the relative importance of features at the level of an input instance, it is especially suitable for clinical applications, where subject-level analysis is required to observe subject-specific patterns of deficit. In this study, Deep SHAP (Lundberg and Lee, 2017) (a SHAP explainer specially geared for DNNs) was applied to explore the relative importance of each unit in the interpretable layer to the classification results. Since the units were trained to conform to the changing trends of acoustic features, their relative importance can reflect the relative contribution of different features of dysarthria (as measured by the four acoustic features) to the diagnosis, thus offering new insights to assist clinicians beyond a binary classification label.

## 3. Experimental Results

### 3.1 Model performance

Table 1 presents the performance of three baselines on dysarthria classification, as well as the accuracy of the proposed model when *w* was set to different values. Several phenomena can be observed from the tables. First, *Baseline 1* yields the lowest accuracy among all evaluated models. While this performance drop can be partly attributed to the difficulty that tree models have with continuous features (Lin *et al.*, 2020), a more likely explanation is that simply fitting a model with the interpretable features fails to capture most of the information available in the speech signal for performing this classification task. In comparison, the other two baselines were trained based on mel-spectrogram. By making use of the abundant information in the input and the majority vote strategy, the other two baselines achieved significantly better classification performance relative to *Baseline 1*. Second, *Baseline 3* offers slightly better performance than *Baseline 2*. This result provides evidence that the bottleneck, even without the interpretable features (as *w* = 1 in this model), is effective in improving the classification accuracy (Yu and Seltzer, 2011). In our experiments, we sweep *w* across a range of values to evaluate the trade-off between model accuracy and representation of the clinically-interpretable layer. For the *w* = 0.9 condition, we observe higher accuracy both at the sample level and speaker level. The improvement in accuracy at the speaker level for the *w* = 0.9 condition over *Baseline 3* was statistically significant based on an upper-tailed paired-samples t-test, t(164)=1.828, *p* < 0.05. The improvement in accuracy at the speaker level for the *w* = 0.9 condition over *Baseline 2* was statistically significant based on an upper-tailed paired-samples t-test, t(164)=1.941, *p* < 0.05. The improvement in accuracy at the speaker level for the *w* = 0.9 condition over *Baseline 1* was statistically significant based on an upper-tailed paired-samples t-test, t(164)=25.29, *p* < 0.05. These findings further advance the idea that it is possible to achieve a model that is both accurate and interpretable through proper optimization (Rudin, 2019). We posit that one of the reasons for the improved performance is that the model is guided towards the correct patterns in data that help distinguish between dysarthric and healthy speech using the interpretable features computed from the complete samples (Ruder, 2017). As a result, a representation that better identifies the phoneme-level difference between dysarthric and healthy speech is learned, and higher overall classification accuracy is achieved. As expected, model accuracy decreases with decreasing *w*.

**TABLE 1.** Model performance for dysarthria classification. The sample-level accuracy is obtained by averaging the cross-validation results at the sample level, and the speaker-level accuracy is obtained by averaging the majority vote cross-validation results at the speaker level. The best accuracy is denoted in bold.

	Sample level (%)	Speaker level (%)
*Baseline 1 (XGBoost)*	—	72.169 ± 10.223
*Baseline 2 (No interpretable layer)*	82.512 ± 3.566	93.531 ± 5.849
*Baseline 3 (Proposed model; w = 1)*	83.031 ± 3.874	93.531 ± 6.473
*w* = 0.9	**83.968 ± 4.390**	**94.708 ± 5.149**
*w* = 0.8	83.629 ± 3.422	94.119 ± 6.200
*w* = 0.7	82.797 ± 3.201	93.532 ± 5.847
*w* = 0.6	81.550 ± 2.338	90.934 ± 4.898

An additional benefit of the joint training strategy is that it also provides a means of better interpreting the model's operation. Each unit in the interpretable layer is correlated to an existing symptom of dysarthria that clinicians currently evaluate perceptually. As *w* continues to decrease, the model pays more attention to learning the interpretable features. When *w* = 0.7, a moderate-to-strong positive correlation is observed between the interpretable features and the speaker-level averaged output of the interpretable layer, as shown in Table 2, while the classification accuracy is still comparable to the baselines. All the correlation coefficients are statistically significant (*p* < 0.0001), which serves as evidence that the model has learned to track the four aspects of dysarthria from the input and represent them in the bottleneck layer. Further reducing *w* to 0.6 improves correlations with the interpretable features, but at the expense of accuracy. The model provides a means of balancing these two tasks, depending on the specific requirements of the application.

**Table 2.** Mean correlation coefficients between the interpretable features and the output of interpretable layer (*w* = 0.7). All the correlation coefficients are statistically significant with *p* < 0.0001, suggesting that the proposed model has learned to track the four features of dysarthria.

	Articulatory precision	CV transition precision	Hypernasality	Vocal quality
PCC	0.585	0.659	0.416	0.332
SCC	0.507	0.533	0.397	0.341

### 3.2 Model interpretation based on SHAP values

The SHAP algorithm was applied to explore the relative importance of the interpretable features to the classification results. For a given sample, the algorithm generates a 4-dimensional vector representing the impact of the four interpretable features on the model's decision. Large positive SHAP value suggests the feature pushes the classification decision toward “Healthy,” whereas a large negative SHAP value indicates the feature pushes the classification decision toward “Dysarthric.” In addition, the mean absolute value of SHAP values provides the global importance of a feature in the model's decision.

**Fig. 2.**
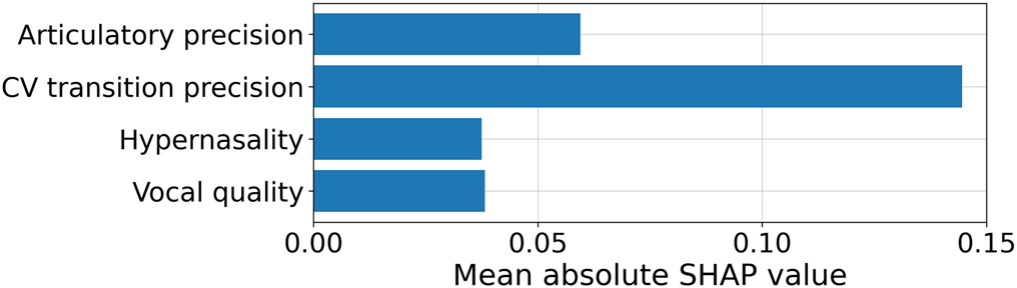
The mean absolute SHAP values computed from the cross-validation (*w* = 0.7). CV transition precision has the largest SHAP value, suggesting the largest contribution to the classification.

We start by performing a global scale (across all speakers) analysis. The mean absolute SHAP values were computed from the test set at each fold of cross-validation (for the *w* = 0.7 model). Then, they were averaged to obtain the global impact of each interpretable feature on the model's decision. The results are shown in Fig. 2.

From Fig. 2, CV transition precision has the largest mean absolute SHAP value, meaning it provides the most discriminative information to help the model classify between dysarthric and healthy samples. In comparison, the contribution of the other three features is relatively small. This finding is aligned with previous clinical studies, as CV boundary regions have been reported to play a key role in the measurement of stop and nasal consonants, and is associated with the common feature of reduced articulatory precision across dysarthrias (Stevens, 2002). In hypokinetic dysarthria, stop consonants have been shown to be especially vulnerable to articulatory degradation (Ackermann and Ziegler, 1991). As for ataxic dysarthria, the uncoordinated movement of the structures of the speech mechanism can cause irregular articulatory breakdown, resulting in distorted vowels and consonants (Love and Webb, 1992). In both cases, the articulatory imprecision can lead to decreased CV transition precision. Considering most of the patients involved in this study come from these two subtypes, it makes sense that the SHAP algorithm selects CV transition precision as an important feature. While there is also reduced articulatory precision in dysarthria, we posit that this is captured by the anomalous CV transitions and therefore the marginal contribution of the articulatory precision is reduced.

**Fig. 3.**
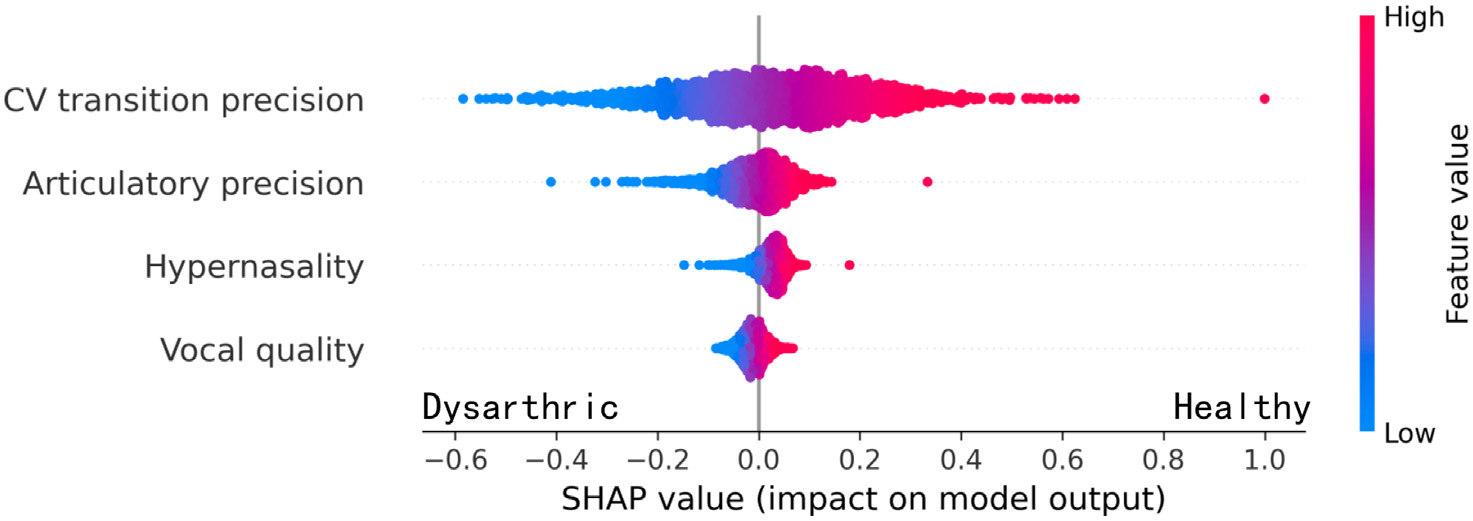
The relationship between the interpretable layer's output and the SHAP values. Larger output values steer the model's decision toward “Healthy,” which aligns with the clinical interpretations of the corresponding interpretable features.

A deeper insight into the features' impact on the model's decision can be obtained by zooming into a single fold of cross-validation and generating a bee swarm plot, as shown in Fig. 3. This analysis shows that the relationship between the interpretable layer's output and the SHAP values are consistent with the clinical interpretations of corresponding interpretable features. For example, higher CV transition precision (as denoted in red) suggests that the CV transitions for the speaker are more like those of healthy controls and drive the model's decision toward “Healthy.” Similarly, higher articulatory precision implies that the coordination of the speaker's articulators is closer to those of healthy controls. Thus, positive SHAP values are generated to push the model's decision toward “Healthy.” The contribution of hypernasality and vocal quality to the prediction is minimal.

**Fig. 4.**
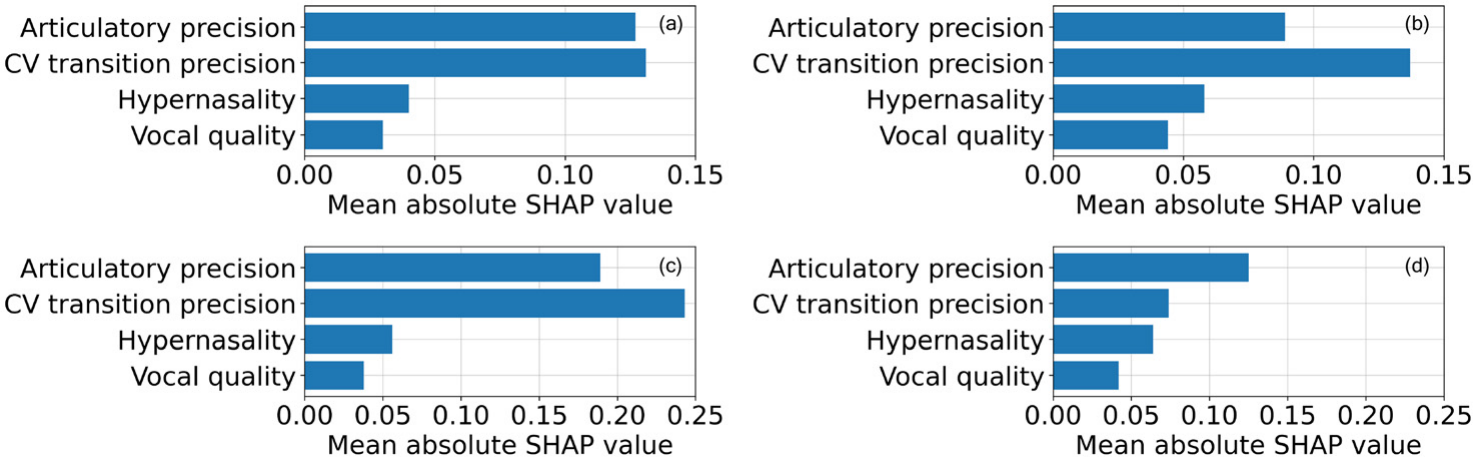
The pattern of deficit obtained from (a) averaging across all speakers with ataxic dysarthria; (b) averaging across all speakers with hypokinetic dysarthria; (c) a typical speaker with ataxic dysarthria; (d) a typical speaker with hypokinetic dysarthria. While both ataxic and hypokinetic speakers generate patterns that emphasize the decreased articulatory precision and control, speakers with hypokinetic dysarthria tend to generate slightly more emphasis on hypernasality and vocal quality, indicating the possible deficits in velopharyngeal and laryngeal functions.

Finally, we show that SHAP can be used to describe subtype-level and subject-level patterns of a deficit. The proposed model was trained using leave-one-subject-out, with a new speaker taken as the test set during each fold. The mean absolute SHAP values were calculated for each test speaker and then averaged at subtype level to generate a set of feature importance values that allow us to compare the pathological patterns across subtypes. Figure 4(a) presents the pattern of deficit obtained from the subset of patients with ataxic dysarthria. CV transition precision and articulatory precision account for most of the contribution, which emphasizes the severity of disturbance in articulatory control, while the lower SHAP values in hypernasality and vocal quality imply a relatively smaller impairment on velopharyngeal and laryngeal function, respectively. In comparison, Fig. 4(b) depicts the pattern of deficit obtained from the subset of patients with hypokinetic dysarthria. This time, the relative importance of interpretable features is distributed more evenly. While articulatory precision and CV transition precision still play an important role, hypernasality and vocal quality account for a larger proportion of contribution, emphasizing the possible breathy and hyper-nasal voice caused by basal ganglia dysfunction. The same analysis can be performed at the individual speaker level. As shown in Figs. 4(c) and 4(d), a typical speaker with ataxic dysarthria displays a pattern of deficit that clearly emphasizes the decreased articulatory control, while a typical speaker with hypokinetic dysarthria demonstrates a distributed pattern of deficit.

## 4. Conclusion

In this study, we introduced a model for dysarthria classification with a clinically-interpretable bottleneck layer. We used a multitask training strategy to jointly learn four interpretable features of dysarthria and the classification label (dysarthria vs control). Then, the SHAP method was adopted to analyze the relative importance of each interpretable feature at a global and individual level. We evaluated the model on a dataset mainly containing speakers from two dysarthria subtypes, predominantly hypokinetic dysarthria. As shown in the results, the proposed model can be flexibly tuned to provide a trade-off between more accurate dysarthria classification or better performance in the prediction of clinically-interpretable features. Interestingly, the bottleneck structure improves the classification performance of the model by constraining the solution space of the DNN, while the introduction of interpretable features steers the model towards known discriminative patterns in data, leading to further improvement. As more attention is paid to interpretability, global-level SHAP analysis reveals CV transition precision to be the most important feature for the model's classification decision; this finding is aligned with previous clinical studies in dysarthria. Finally, the subtype-level and individual speaker-level SHAP analysis identify specific patterns from different dysarthric speakers. This has the potential to help clinicians better understand binary decisions made by clinical speech machine learning models.

The current model uses SHAP values for interpretation. It should be noted that this is but one approach for assessing the internal workings of a model with strengths and limitations. First, the SHAP method implementation used in this study only provides an approximation of the true Shapley values. Furthermore, the SHAP method can be sensitive to the final DNN solution to which the model converges. We attempted to alleviate the variability by training the model multiple times and taking the mean SHAP results. Finally, the interpretable features covary and this interaction can challenge the interpretability of the model. Future work will focus on further advancing the interpretability of clinical speech models by overcoming some of these limitations.

The sample size used to train the model was relatively small and the dysarthric group did not include all dysarthria subtypes. Previous work has shown that estimates of accuracy on small datasets tend to be overoptimistic (Berisha *et al.*, 2021). In our work, as the test set was used to determine when to stop training the three baselines and our proposed model, it is likely that the estimates of accuracy are positively biased. However, in this work, we were not directly interested in the absolute estimates of accuracy but rather their relative difference; previous work has shown that positively biased estimates of accuracy can be reliably used to compare model performance (Wainer and Cawley, 2021). Future work will require the estimation of accuracy on a much larger dataset or a prospective validation of the model. In addition, prior to prospective validation on all dysarthria subtypes, future work should also focus on collecting a more diverse training set that includes representation from all dysarthria subtypes.
